# Long-term prognosis of chronic cough: a prospective, observational cohort study

**DOI:** 10.1186/s12890-017-0496-1

**Published:** 2017-11-21

**Authors:** Heikki O. Koskela, Anne M. Lätti, Minna K. Purokivi

**Affiliations:** 1 Unit for Medicine and Clinical Research, Pulmonary Division, Kuopio University Hospital, Puijonlaaksontie 2, 70120 Kuopio, Finland; 2 School of Medicine, Institute of Clinical Sciences, Faculty of Health Sciences, University of Eastern Finland, Kuopio, Finland

**Keywords:** Cough, Chronic cough, Esophageal reflux disease, Asthma, Rhinitis

## Abstract

**Background:**

The long-term prognosis of chronic cough and its determinants need to be clarified.

**Methods:**

This is a prospective, observational cohort study. Eighty-nine unselected subjects with chronic (> 8 weeks’ duration) cough were carefully investigated: Clinical examination, symptom questionnaire, Leicester Cough Questionnaire (LCQ), skin prick tests, ambulatory peak expiratory flow monitoring, spirometry before and after 0.4 mgs of salbutamol, exhaled nitric oxide concentration measurement, hypertonic saline cough provocation test, and histamine bronchial provocation test. After five years, a letter was sent to the subjects containing questions about continuation of cough, smoking, indoor exposures, presence of co-morbidities, and current medication. It also contained LCQ and Cough Clinic diagnostic questionnaire. Sixty-eight subjects (76%) responded.

**Results:**

At five years, continuing regular cough was present in 31 (46%) of the subjects and continuing impairment in cough-related quality of life (less than 1.3 points’ improvement in LCQ) in 32 (47%). Continuing regular cough was associated with presence of chronic rhinitis or esophageal reflux disease, baseline mild airway responsiveness to histamine, and baseline strong cough responsiveness to hypertonic saline. Continuing impairment in cough-related quality of life was associated with high body mass index, absence of atopy, absence of pets, and high number of background disorders (esophageal reflux disease, asthma, or chronic rhinitis).

**Conclusions:**

Almost half of subjects with chronic cough suffered of the disorder at five years from initial assessment. Several possible determinants of poor prognosis could be identified.

## Background

Cough is a clinically highly important symptom. It is the most common reason why people seek medical attention [1, 2] and has a strong negative effect on the quality of life (QoL) [3, 4].This is especially true about chronic cough, defined as cough which lasts longer than eight weeks [5, 6]. Knowledge about the long-term prognosis of chronic cough and its determinants would be valuable to guide the management of subjects with chronic cough.

To the best of our knowledge there is only one study about the long-term prognosis of chronic cough [7]. That study included 42 subjects with unexplained (idiopathic) chronic cough who had attended a specialist cough clinic seven years earlier. At the time of re-assessment, the cough was unchanged or had worsened in 60% of the patients. The population of that study can be regarded as selected because one or more treatable causes of chronic cough can usually be identified [5, 6]. Therefore, it is necessary to investigate the long-term prognosis and its determinants in a more representative group of subjects with chronic cough.

## Methods

**Fig. 1:**
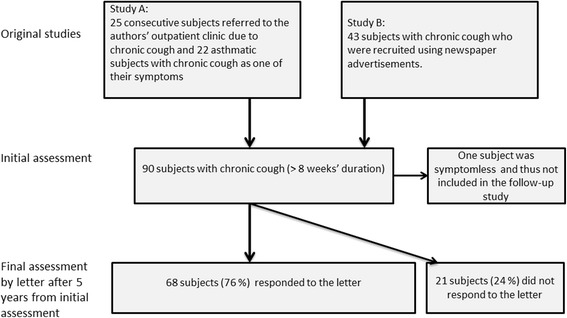
The flow chart of the study. Study A, see reference [8]. Study B, see reference [9]

This is a prospective, observational cohort study. The subjects had participated either of two clinical studies investigating the clinical utility of hypertonic saline cough provocation test. This was defined as the ‘initial assessment’ in the present study. The first of these studies [8] included 25 consecutive subjects who had been referred to a tertiary referral clinic due to chronic cough (defined as >8 weeks’ duration) and 22 asthmatic subjects with chronic cough as one of their symptoms. The second study [9] included 43 subjects with chronic cough who were recruited using newspaper advertisements. One of the 90 subjects thus recruited was symptomless during the initial assessment and was therefore not included in the follow-up study. Therefore, 89 subjects with chronic cough were included in the follow-up study (Fig. 1).

The following examinations had been carried out in all subjects at initial assessment: Clinical examination, symptom questionnaire, Leicester Cough Questionnaire (LCQ) [10], skin prick tests, ambulatory peak expiratory flow monitoring, spirometry before and after 0.4 mgs of salbutamol, exhaled nitric oxide concentration measurement, hypertonic saline cough provocation test [8], and histamine dosimetric bronchial provocation test [11]. The response to histamine was expressed as response-to-dose ratio (RDR): The maximal percentage fall in forced expiratory volume in one second related to the final dose of histamine. Further details about the examinations are presented in the original publications [8, 9]. The hypertonic saline test and LCQ are described below. All subjects were offered the option to visit a staff respiratory physician after the original studies for treatment of the chronic cough. However, this was not part of the study protocols and the therapy was not standardized.

### Hypertonic saline cough provocation test [8]

First, spirometry was performed. Then the subjects inhaled 0.4 mg of salbutamol to prevent bronchoconstriction and 15 min later the spirometry was repeated. After that the subject inhaled isotonic phosphate-buffered saline for two minutes via a high-output ultrasonic nebuliser (DeVilbiss Ultraneb 3000, Sunrise Medical Ltd., West Midlands, UK), using tidal breathing. The coughs occurring during the inhalation and two minutes after it were manually counted. The number of these coughs was subtracted from the coughs provoked by each hypertonic solution. Subsequently, they inhaled hypertonic phosphate-buffered saline solutions with osmolalities of 0.6, 0.9, 1.2, 1.5, 1.8 and 2.1 Osm/kg. The challenge was stopped if 15 or more cumulative coughs were recorded. The cough response to hypertonic saline was expressed as coughs-to-dose-ratio (CDR): The cumulative number of provoked coughs divided by the final osmolality inhaled.

### Leicester cough questionnaire

The Leicester Cough Questionnaire (LCQ) is a 19-item validated, repeatable and responsive questionnaire consisting of physical, psychological and social domains. Answers are graded on 7-point Likert scale which gives a total score ranging from 3 to 21. A small score indicates poor cough-related QoL and an increase in score indicates an improvement in the QoL [10]. The minimal important difference of LCQ total score is 1.3 points [12].

### The follow-up study

After five years from the initial assessment, a letter was mailed to the subjects containing questions about continuation of cough, smoking, indoor exposures, current medication, and presence of co-morbidities. Especially, the presence of the well-known chronic cough background disorders, namely esophageal reflux disease, asthma, and chronic rhinitis, was asked. The letter also contained LCQ and the Cough Clinic diagnostic questionnaire [13]. The latter is a 16-item questionnaire to assess the most probable background disorder of chronic cough. If a subject did not respond to the first letter, it was sent to the subject once more.

### The outcome measures

There were two outcome measures. One was continuing regular (on most days of the week) cough at follow-up. The second was continuing impairment in cough-related QoL, defined as less than 1.3 point’s increase in LCQ total score when comparing the initial assessment and the follow-up assessment.

### Statistical analysis

The descriptive data is expressed as means and standard deviations. However, the distribution of RDR to histamine and CDR to saline differed significantly from normal distribution (*p* < 0.01, Kolmogorov-Smirnov test). Therefore, logarithm transformed values were used for parametric statistical analysis and geometric means and standard deviations are shown. The results are expressed as means and 95% confidence intervals. The bivariate associations of various features with the outcomes were assessed utilizing Student’s T-test, Chi-square test, and binary logistic regression analysis. Very rare features (presented by less than 10 subjects), were not analyzed. The multivariate associations were assessed utilizing binary logistic regression analysis with backward directed stepwise process. Analyses were performed using SPSS version 22 for the personal computer (SPSS, Inc. Chicago, Illinois, USA).

## Results

Sixty-eight out of the 89 subjects (76%) responded to the follow-up letter and returned the questionnaires (Table 1). One subject had died. Comparing the non-responders with the responders there were no statistically significant differences in sex distribution, age, BMI, and duration of cough. However, the LCQ total score at initial assessment was lower in the non-responders (12.4 (10.7–14.1) vs. 14.4 (13.6–15.2), *p* = 0.02).

**Table 1:** The characteristics of the 68 subjects who responded to the follow-up letter

Age, years	59 ± 11
Female sex	51 (75%)
Body mass index at initial assessment, kg/m^2^	27.6 ± 4.3
Atopic subjects	28 (41%)
Ex-smokers	27 (40%)
Current smokers	1 (2%)
Passive smoking	6 (9%)
Pet ownership	24 (35%)
Home damaged by dampness	8 (12%)
Duration of cough at initial assessment, years	6.0 ± 6.3
Presence of an autoimmune disease	17 (25%)
with hypothyroidism	13
Presence of chronic rhinitis	32 (47%)
with nasal corticosteroids	21
with antihistamines	9
Presence of doctor’s diagnosis of asthma	34 (50%)
with inhaled corticosteroids	27
Presence of esophageal reflux disease	17 (25%)
with proton pump inhibitors	6
Responsiveness to histamine airway challenge at initial assessment, RDR, %/mg ^a^	10 ± 5
Responsiveness to hypertonic saline cough provocation test at initial assessment, CDR, coughs/Osm/kg ^a^	4.7 ± 7.1
Mean daily mean peak flow variation at initial assessment, percent	6.1 ± 4.3
FEV_1_ at initial assessment, percent of predicted ^b^	96 ± 14
Improvement in FEV_1_ after salbutamol at initial assessment, percent	4.3 ± 4.7
Exhaled nitric oxide concentration at initial assessment, ppm	21 ± 19

Data is expressed as means ± SDs or as percentages. ^a^Geometric means and SDs. ^b^The predicted values are from reference [24]. FEV_1_ = forced expiratory volume in one second, RDR = Response-to-dose ratio; CDR = coughs-to-dose ratio

**Fig. 2:**
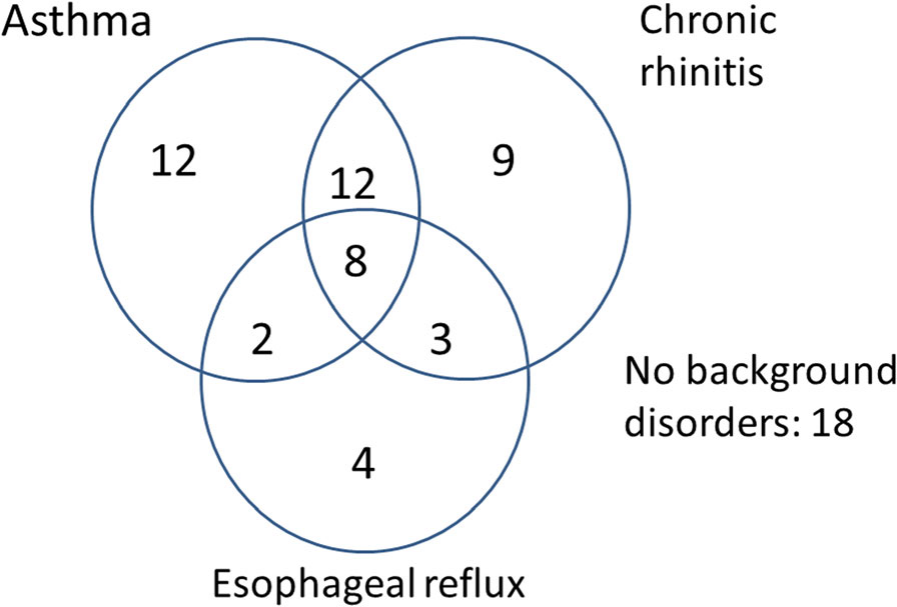
A Venn diagram showing the co-existence of chronic cough background disorders in the 68 subjects who responded to the follow-up letter

According to the subject’s own report, asthma was the most common background disorder with many reporting of several background disorders (Table 1, Fig. 2). Eighteen subjects (26%) did not report of any background disorder. According to Cough Clinic diagnostic questionnaire, the most probable background disorder of chronic cough was chronic rhinitis in 44 subjects, esophageal reflux disease in 21, and asthma in 7. None of the subjects used angiotensin-converting enzyme inhibitors.

At follow up, regular cough continued in 31 out of the 68 subjects (46%). Continuing impairment in cough-related QoL was demonstrated in 32 subjects (47%). Naturally, there was marked overlapping between the two groups (*p* < 0.001). However, there were also eight subjects with continuing regular cough but with a significant improvement in cough-related QoL. There were also another eight subjects with continuing impairment in cough-related QoL but who reported that the regular cough had stopped.

In the whole group of 68 patients the mean LCQ total score had increased significantly (14.4 (13.6–15.2) at initial assessment and 15.8 (14.9–16.8) at follow-up, *p* = 0.004). Naturally, the increase in LCQ total score was greater in subjects who did not cough regularly at follow-up than in those who did (from 14.8 (13.8–15.8 to 18.1 (17.2–19.0), *p* < 0.001, and from 13.9 (12.7–15.2) to 13.2 (11.8–14.5), *p* = 0.2, respectively).

**Fig. 3:**
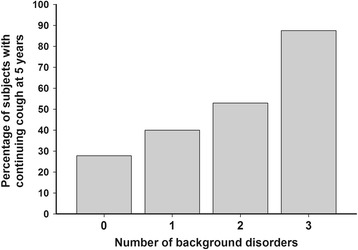
The proportion of subjects who suffered from continuing regular cough at five years’ follow-up related to the number of self-reported background disorders (esophageal reflux disease, asthma, or chronic rhinitis). Eighteen subjects reported no background disorders, 25 reported one disorder, 17 reported two disorders, and 8 reported three disorders. *P* = 0.03 between the groups (Chi-square test), *p* = 0.007 for the trend between the number of background disorders and continuing cough (univariate logistic regression analysis)

There were several features that showed at least suggestive (*p* < 0.10) bivariate association with continuing regular cough at follow-up: High body mass index (BMI), low histamine RDR, and high saline CDR. Furthermore, several background disorder-related features showed an association with the continuing cough: Self-reported chronic rhinitis, self-reported esophageal reflux disease, presence of either of those, and number of background disorders (Fig. 3). Of them, presence of either rhinitis or reflux showed the closest association with the outcome and was therefore included in the multivariate analysis. The results of the multivariate analysis about continuing cough at follow-up are expressed in Table 2.

**Table 2:** The determinants of continuing cough at five years from initial assessment

	Adjusted OR	95% CI	*P* value
Chronic rhinitis or reflux disease	8.0	2.2–29	0.001
RDR to histamine at initial assessment	0.19^a^	0.06–0.65	0.008
CDR to hypertonic saline at initial assessment	2.4^b^	1.0–5.6	0.05

^a^Adjusted OR calculated per one %/mg. ^b^Adjusted OR calculated per one cough/Osm/kg. RDR = response-to-dose ratio; CDR = coughs-to-dose-ratio

**Fig. 4:**
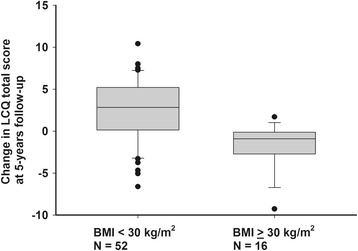
The change in Leicester cough questionnaire (LCQ) total score at follow-up in relation to the subjects’ body mass index (BMI) at initial assessment. An increase in LCQ total score indicates an improvement in the cough-related quality of life. The lower boundary of the box indicates the 25th percentile, a line within the box marks the median, and the higher boundary indicates the 75th percentile. The error bars above and below the box indicate the 90th and 10th percentiles. *P* < 0.001 between the groups (unpaired T-test)

There were also several features that showed at least suggestive (*p* < 0.10) bivariate association with continuing impairment in cough-related QoL: Absence of pets, absence of atopy, high age, high BMI (Fig. 4), high initial LCQ total score, and high saline CDR. Again, there were also several background disorder-related features that showed an association with this outcome: Self-reported asthma, self-reported esophageal reflux disease, presence of either of those, and number of background disorders. Of them, the number of background diseases showed the closest association with the outcome and was therefore included in the multivariate analysis. The results of the multivariate analysis about the factors associated with continuing impairment in cough-related QoL are expressed in Table 3.

**Table 3:** The determinants of continuing impairment in cough-related quality of life (< 1.3 points increase in Leicester Cough Questionnaire) at five years from initial assessment

	Adjusted OR	95% CI	*P* value
Body mass index at initial assessment	1.5 ^a^	1.2–2.0	0.001
Atopy	0.19	0.04–0.84	0.03
Pet at home	0.27	0.06–1.2	0.08
Number of background disorders	2.2 ^b^	0.95–4.9	0.06

^a^Adjusted OR calculated per one kg/m^2^. ^b^Adjusted OR calculated per one background disorder

Several variables did not show any association with the outcomes including sex, duration of cough at initial assessment, exhaled nitric oxide level, peak flow variation, Cough Clinic diagnostic questionnaire-defined most probable cause of cough, and absence of adequate medication for background disorders.

## Discussion

The present study showed that almost half of the subjects with chronic cough continue coughing regularly after five years. Further, roughly the same proportion of subjects experience continuing impairment in cough-related QoL at that time-point. Several factors which showed associations with these outcomes could be identified.

The prognosis of chronic cough in the present study was slightly better than in the previous study about this issue [7]. In that study, cough was unchanged or had worsened in 60% of the patients at seven-year follow-up. This small discrepancy can best be explained by the differences in the populations. The former study included only patients with unexplained (idiopathic) chronic cough. The present study included patients with various typical cough background disorders as well as patients with idiopathic cough, and probably better represents subjects with chronic cough in general. The prevalence of autoimmune diseases was high in the present group, a feature which has this far connected to the idiopathic form of chronic cough [14]. The majority of the subjects with asthma and chronic rhinitis were adequately medicated with local corticosteroid preparations or antihistamines. On the contrary, the majority of the subjects with reflux disease did not use proton pump inhibitors suggesting undertreatment. However, monotherapy with these drugs is found to be inefficient in reflux-associated cough [15]. Thus, the present population probably well reflects real-life patients also with respect to current medications.

Most of the subjects with continuing regular cough also suffered from continuous impairment in cough-related QoL. However, there were also several subjects who reported only either one. Furthermore, continuing regular cough and continuous impairment in cough-related QoL were associated with different determinants. Thus, they can be regarded as complementary outcomes, measuring different modalities in chronic cough. There seems to be subjects whose continuous cough may not disturb them as much as earlier and subjects whose cough has turned from continuous to periodic but who still suffer from significant impairment in cough-related QoL.

Unlike the previous study [7], the present study could identify several factors which were associated with the outcomes. An increase in the number of background disorders (esophageal reflux disease, asthma, and rhinitis) was associated with both continuing regular cough and continuing impairment in cough-related QoL. The type of background disorder may be of lesser importance: Cough Clinic diagnostic questionnaire-defined most probable cause of chronic cough [13] showed no association with the outcomes. There are potential flaws with this questionnaire. Firstly, as it suggests one background disorder in all subjects, the idiopathic form of chronic cough will not be recognized. Secondly, as it usually suggests just one disorder, the other relevant background disorders remain unrecognized. The present results encourage treating all background disorders in a subject with chronic cough, not just the most evident one.

Strong airway responsiveness to histamine predicted cessation of regular cough whereas strong cough responsiveness to hypertonic saline was associated with continuing regular cough. Strong responsiveness to histamine indicates that the subject’s airway smooth muscle is prone to contract. Airway smooth muscle contraction causes a mechanical stimulation of the airway rapidly adapting receptors, thus inducing cough [16]. Hyperresponsiveness to histamine may indicate that a subject suffers from this kind of cough. With current asthma medications the attacks of smooth muscle contractions can usually be eliminated, probably resulting to disappearance of this type of cough. Indeed, it has been shown that airway hyperresponsiveness to histamine strongly predicts healing of cough in response to inhaled corticosteroid [9]. Strong cough responsiveness to hypertonic aerosols, in turn, probably indicates a dysfunction of the cough reflex arch [8, 16] which currently lacks efficient therapeutic options.

The presence of atopy predicted improvement in cough-related QoL. Atopy, a marker of allergic disorders, may also reflect a drug-sensitive mechanism of chronic cough, similarly to the airway responsiveness to histamine. The association between pets at home and improvement in cough-related QoL may well represent a selection bias. The subjects in whom chronic cough disappears may be more prone to acquire pets than subjects with continuous cough. However, there are reports showing that pet exposure in early life might prevent the development of respiratory symptoms [17, 18].

Finally, a strong potential determinant of continuing impairment in cough-related QoL was identified, namely obesity. Indeed, the subjects fulfilling the current definition of obesity [19] (BMI > 30 kg/m^2^) showed virtually no improvement in LCQ total score and a marked decrease was documented in many. High BMI remained a strong determinant of continuing impairment in cough-related QoL even in the multifactorial analysis including the reflux disease. Thus, reflux disease cannot explain the association. There are population-based studies reporting an association between obesity and chronic cough [20, 21]. Obesity is also associated with chronic obstructive pulmonary disease and asthma, and in asthma it is associated with poor response to corticosteroids. Adipose tissue is an important source of adipokines. The balance between pro- and anti-inflammatory adipokines contributes to the physiological maintenance of the lung microenvironment [19, 22]. It may be hypothesized that obesity-related alterations in this balance could contribute to the appearance and prolongation of cough.

There are several limitations in the present study. The information about the background disorders at baseline was insufficient and at follow-up it was relied on the subjects’ own assessment and not on a current clinical diagnosis. However, in Finland, the doctor’s diagnosis of asthma is always based on objective evidence of reversible airway obstruction, according to the Finnish Social Insurance Institution criteria [23]. At the moment there are no diagnostic tests to assure that a subject’s cough is due to esophageal reflux [15] and the diagnosis of chronic rhinitis can only be based on subjective symptoms. The population in the present study consists of three groups from two studies which may make the interpretation of the results difficult. However, by combining the three groups we gained a representative sample consisting of cough subjects with different etiologies and degrees of severity. A large variation in characteristics was statistically advantageous to define features that correlate with prognosis.

There were 21 subjects who did not respond to the follow-up letter. They differed from the rest of the subjects only with respect to the initial LCQ total score. However, the initial LCQ showed no independent association with the outcome measures. Therefore, we assume that the non-responders have not skewed the results significantly. One may also criticize the use of multivariate analysis in a relatively small population. However, there were several possible determinants of the outcomes and we wanted to define the most important ones. We fully acknowledge that the present study reports just associations, not causalities. The lack of standardized management and drug compliance markers can also be considered as weaknesses. However, this was not a clinical drug trial but an observational cohort study. In any case, the management mainly followed the current guidelines and the setting probably well reflects the real-life situation.

## Conclusions

Almost half of subjects with chronic cough still suffer from the disorder at five years from initial assessment. A subject can experience impaired cough-related QoL even if the cough is not regular anymore. Thus, an improvement in a validated QoL questionnaire may be the most relevant outcome variable in everyday clinical practice. Further, all background disorders that may be contributing to cough should be effectively managed, not just the most evident one. Indicators of possible medication-sensitive cough like atopy and airway hyperresponsiveness to histamine should be recognized. Strong cough responsiveness to hypertonic aerosol may be an indicator of dysfunction of the cough reflex arch and poor prognosis. Finally, obesity seems to be strongly associated with poor prognosis in chronic cough and an intervention for weight loss is a promising therapeutic option to be evaluated in the future.

## Abbreviation

BMI: Body mass index
CDR: Coughs-to-dose-ratio
LCQ: Leicester Cough Questionnaire
QoL: Quality of life
RDR: Response-to-dose ratio
